# Biomarkers in Patients With Acute Ischaemic Stroke Undergoing Thrombectomy Compared With Controls: A Pilot Study

**DOI:** 10.1111/aas.70391

**Published:** 2026-11-08

**Authors:** Richard Vithal, Andreas Krönlein, Ali El‐Merhi, Amar Chandan, Hilda Green, Patrik Martner, Christina Biörserud, Helena Odenstedt Herges, Henrik Zetterberg, Alexandros Rentzos, Katarina Jood, Magnus Pettersson, Jaquette Liljencrantz, Linda Block

**Affiliations:** ^1^ Department of Anesthesiology and Intensive Care, Institute of Clinical Sciences, Sahlgrenska Academy University of Gothenburg Gothenburg Sweden; ^2^ Department of Anaesthesia and Intensive Care, Region Västra Götaland Sahlgrenska University Hospital Gothenburg Sweden; ^3^ Department of Surgery, Institute of Clinical Sciences, Sahlgrenska Academy University of Gothenburg Gothenburg Sweden; ^4^ Department of Surgery, Region Västra Götaland Sahlgrenska University Hospital Gothenburg Sweden; ^5^ Department of Psychiatry and Neurochemistry, Institute of Neuroscience and Physiology, Sahlgrenska Academy University of Gothenburg Mölndal Sweden; ^6^ Clinical Neurochemistry Laboratory Sahlgrenska University Hospital Mölndal Sweden; ^7^ Department of Neurodegenerative Disease UCL Institute of Neurology Queen Square, London UK; ^8^ UK Dementia Research Institute at UCL London UK; ^9^ Hong Kong Centre for Neurodegenerative Diseases, Clear Water Bay Hong Kong China; ^10^ Wisconsin Alzheimer's Disease Research Centre, University of Wisconsin School of Medicine and Public Health University of Wisconsin‐Madison Madison Wisconsin USA; ^11^ Department of Radiology, Institute of Clinical Sciences, Sahlgrenska Academy University of Gothenburg Gothenburg Sweden; ^12^ Department of Radiology, Region Västra Götaland Sahlgrenska University Hospital Gothenburg Sweden; ^13^ Department of Clinical Neuroscience, Institute of Neuroscience and Physiology, Sahlgrenska Academy University of Gothenburg Gothenburg Sweden; ^14^ Department of Neurology, Region Västra Götaland Sahlgrenska University Hospital Gothenburg Sweden; ^15^ Department of Statistics, Institute of Medicine and Public Health, Sahlgrenska Academy University of Gothenburg Gothenburg Sweden

**Keywords:** anaesthesia, biomarkers, cerebral ischaemia, critical care, glial fibrillary acidic protein

## Abstract

**Background:**

Detection of cerebral ischaemia is challenging in patients who are anaesthetised, sedated or have reduced levels of consciousness, as neurological examination is difficult or even impossible to assess. Furthermore, as some patients cannot be transported for radiology, for example during surgery, a reliable blood biomarker for detecting acute cerebral events would be of considerable clinical value. We aimed to compare levels of brain injury biomarkers glial fibrillary acidic protein (GFAP), neurofilament light chain (NfL), and total tau (tau) in patients with acute ischaemic stroke (AIS) undergoing endovascular thrombectomy under general anaesthesia or sedation to controls undergoing non‐cerebral surgery under general anaesthesia. These biomarkers were chosen because previous studies have demonstrated their potential as blood biomarkers of acute cerebral injury and ischaemic stroke.

**Methods:**

In this prospective, observational pilot study, conducted at the Sahlgrenska University Hospital, Gothenburg, Sweden, between 2021 and 2024, GFAP, NfL, and tau were analysed using the Simoa Neurology 4‐plex assay, before thrombectomy or surgery and at 2‐ and 24 h thereafter.

**Results:**

Blood samples from 21 patients with AIS and 19 controls were analysed. GFAP concentrations in patients with AIS increased significantly from baseline to 24 h (median 301.0 → 1443.0 pg/mL; *p* = 0.001) and were higher than in controls at all time points (*p* < 0.01). In the controls there was no significant increase in GFAP from baseline to 24 h (*p* = 0.449). NfL and tau increased significantly in patients with AIS (*p* = 0.001 and 0.002) but also in the controls (*p* = 0.001 and 0.047).

**Conclusion:**

GFAP and NfL concentrations were higher in patients with acute ischaemic stroke than in surgical controls at all sampling time points, whereas tau differed only after 24 h. Unlike NfL and tau, GFAP did not increase significantly in controls, suggesting that it may be less influenced by anaesthesia and surgery. These findings support further evaluation of GFAP as a potential biomarker of acute cerebral injury in perioperative and critical care settings.

**Editorial Comment:**

A biomarker of ischaemic cerebral injury could be attractive to support treatments to protect brain health during anaesthesia and surgery. In a pilot study, glial fibrillary acidic protein emerged as a potential candidate as it was increased in patients undergoing endovascular thrombectomy for large‐vessel ischaemic stroke compared to anaesthetised patients undergoing non‐cerebral surgery.

**Trial Registration:**

ClinicalTrials.gov identifier: NCT03919370

## 1 Introduction

Every day, critically ill patients are admitted to intensive care units (ICUs) with life‐threatening conditions. Acute cerebral events such as ischaemia or haemorrhage are a direct threat in stroke and brain injury, but also a potential threat following systemic hypotension during surgery and anaesthesia [1]. Critically ill patients suffering from sepsis, cardiac failure and coagulation disorders are also at risk for cerebral ischaemia [2]. Prompt detection and acute management of cerebral events are essential to prevent brain injury, as the brain is highly vulnerable to insufficient oxygen supply [3]. The most readily accessible method for detecting acute cerebral events is the clinical neurological examination of a fully awake patient. However, many critically ill patients are sedated or anaesthetised, making accurate neurological assessment difficult or impossible. Furthermore, as some patients cannot be transported for radiology, for example during surgery, a reliable blood biomarker for detecting acute cerebral events, comparable to troponin T or I in cardiac ischaemia, would be of considerable clinical value.

For a biomarker to effectively detect an acute cerebral event in the clinically complex ICU setting, several criteria must be met. The biomarker must be highly brain‐specific, be unaffected by anaesthetic drugs, surgery, other invasive interventions and trauma, cross the blood–brain barrier, and be rapidly detectable in peripheral blood [4]. There are several potential candidates, including neurofilament light chain (NfL), total tau (tau), and glial fibrillary acidic protein (GFAP) [5, 6]. NfL is primarily present in myelinated axons [7, 8, 9], and tau is predominantly present in unmyelinated axons [10, 11], in both the central and peripheral nervous systems. However, tau is also expressed in the kidney, heart, and liver, which may limit its usefulness as a brain injury biomarker in a surgical setting, even though there is limited research on this topic [12].

Since both tau and NfL are present in the peripheral nervous system, theoretically, they could be released or leaked into the bloodstream during any surgical procedure, but there is also the possibility of anaesthesia and surgery itself causing neurotoxicity [13].

GFAP is primarily expressed in astrocytes of the central nervous system and has been reported as the most brain‐specific of the available brain injury biomarkers [5]. It is also suggested to be sensitive in acute cerebral ischaemia but not to chronic brain damage [14]. Furthermore, it may potentially aid in the discrimination between haemorrhagic and ischaemic stroke as well as for prognostication of outcome [15, 16, 17, 18].

Previous research has demonstrated that haemorrhagic stroke induces a stronger release of GFAP than acute cerebral ischaemia. However, by repeated sampling, bleeding and ischaemia can be differentiated as described in the proof‐of‐concept article by Mattila et al. [19] Nonetheless, a validated blood biomarker for the detection of ongoing cerebral injury during anaesthesia and critical illness remains lacking [20].

As a clinical model we enrolled patients with acute ischaemic stroke (AIS) undergoing cerebral thrombectomy, a clinical intervention performed in sedation or under general anaesthesia. To enable comparison, we also enrolled patients undergoing non‐cerebral surgery under general anaesthesia, who served as controls. The control group was included to allow us to investigate if anaesthesia and surgery per se could cause a release of brain injury biomarkers into the blood, which could obscure the results in anaesthetised patients undergoing surgery or other interventions.

Our primary aim was to compare the plasma concentrations of GFAP, NfL, and tau in patients with AIS undergoing cerebral thrombectomy performed in sedation or under general anaesthesia to those of control patients undergoing non‐cerebral surgery under general anaesthesia.

We hypothesised that the brain injury biomarkers GFAP, NfL, and tau would be rapidly released into the bloodstream during AIS, but not in control patients.

The secondary aim was to map the temporal changes in plasma levels of GFAP, NfL, and tau during AIS. The hypothesis was that a significant elevation would be observed from the pre‐intervention phase through 24 h after the intervention, compared to the controls.

## 2 Methods

### 2.1 Setting

This prospective observational pilot study was conducted at Sahlgrenska University Hospital, Gothenburg, Sweden, between September 2021 and November 2024, and is one of several studies constituting the project Cerebral Ischaemia Detection using Artificial Intelligence (CIDAI), which aims to develop methods for detecting cerebral ischaemia in patients with reduced consciousness or under sedation. The CIDAI study protocol was published in June 2020 [21] and the protocol was supplemented with detection of cerebral ischaemia with blood biomarkers in October 2020. This study was approved by the Swedish Ethical Review Authority (Dnr 2020‐00169) on March 10, 2020, with amendment 2020‐05122 on October 31, 2020. The study protocol adheres to the latest version of the Declaration of Helsinki. It is registered on ClinicalTrials.gov (NCT03919370). This manuscript adheres to the Strengthening the Reporting of Observational Studies in Epidemiology (STROBE) guidelines [22].

### 2.2 Groups, Patients and Procedures

#### 2.2.1 Both Groups

As the study setup, including blood sampling and sample management, required the presence of a research assistant or PhD student, eligible patients were identified when research resources were available. Documented consent was obtained for included study participants in accordance with the regulations of the Swedish ethical review authority. Inclusion criteria included adults aged > 18 years who were assessed by the attending neurologist to undergo endovascular thrombectomy for the AIS group or scheduled to undergo mixed abdominal surgery under general anaesthesia for the control group, and who consented to participate in the study. Patients were excluded if blood samples could not be obtained or if consent was withdrawn.

All patients underwent continuous invasive arterial monitoring throughout the procedure. In the control cohort, blood pressure management aimed to maintain mean arterial pressure (MAP) as close as possible to each patient's pre‐anaesthetic baseline. In patients undergoing thrombectomy, in accordance with guidelines, MAP was targeted above 100 mmHg, with systolic blood pressure targets of < 220 mmHg.

#### 2.2.2 Acute Ischaemic Stroke Group

Patients were recruited from the hospital stroke chain; research staff were alerted whenever a patient with AIS was directed from the initial in‐hospital assessment to treatment with endovascular thrombectomy. If the patient could not consent due to their condition, next of kin were consulted.

#### 2.2.3 Endovascular Thrombectomy and Anaesthesia Procedures for the Acute Ischaemic Stroke Group

Patients eligible for endovascular thrombectomy were anaesthetised or sedated as per hospital protocol; the study did not influence this. General anaesthesia was induced with propofol, ketamine and remifentanil and maintained with sevoflurane and remifentanil. Rocuronium or suxametonium was used as a muscle relaxant. For sedation, patients were treated with remifentanil only. All patients had monitoring of vital parameters, including continuous invasive arterial blood pressure. The endovascular thrombectomy procedure is well‐documented for its efficacy and safety, having been extensively studied for over a decade [23, 24, 25]. Contrast extravasation during thrombectomy was assessed continuously by the interventional neuroradiologist using fluoroscopic imaging throughout the procedure. In addition, as per clinical routine, all thrombectomy patients underwent dual‐energy computed tomography 12–24 h after thrombectomy to assess postprocedural intracranial haemorrhagic complications.

#### 2.2.4 Control Group

Patients were recruited from the hospital's surgical schedule without sex‐ or age‐matching and included at the preoperative assessment. Exclusion criteria were atrial fibrillation and previous neurological conditions.

#### 2.2.5 Surgery and Anaesthesia Procedures for the Control Group

All patients had monitoring of vital parameters, including continuous invasive arterial blood pressure. Anaesthesia was induced with propofol and fentanyl or propofol and remifentanil. Maintenance was obtained with sevoflurane combined with fentanyl boluses or with a continuous remifentanil infusion. All patients received the muscle relaxant rocuronium, underwent endotracheal intubation, and were mechanically ventilated. All patients were followed up with the National Early Warning Score (NEWS) for 48 h; none showed signs of cerebral injury postoperatively. The surgery consisted of robot‐assisted laparoscopic prostatectomy, hysterectomy, hemicolectomy, and kidney resection, as well as open hemicolectomy and kidney pyeloplasty. For further details on the control group, refer to the 2025 publication [26].

### 2.3 Sample Collection and Biomarker Analysis

Blood samples for GFAP, NfL, and tau were collected at three time points to investigate their temporal profile. The sampling time points were immediately before anaesthesia onset (before the intervention), 2 h after thrombectomy/surgery, and finally, 24 h after thrombectomy/surgery.

The research staff collected baseline samples from an arterial line; subsequent samples were drawn from a venous needle or venous line. Samples were collected in plasma tubes, centrifuged, aliquoted, and stored at −80°C in a temperature‐controlled freezer until the lab could analyse them. All samples were analysed at the Clinical Neurochemistry Laboratory of the Institute of Neuroscience and Physiology, Sahlgrenska Academy, University of Gothenburg, Mölndal Campus, Sweden.

The plasma concentrations of GFAP, NfL and tau were measured using the Simoa (Single molecule array) Neurology 4‐plex assay kit (Quanterix, Billerica, MA) on an HD‐X Analyser. Simoa is an automated immunoassay platform with femtoliter reaction chambers that utilises anti‐analyte antibodies conjugated to magnetic beads with biotinylated antibodies to create a digitally detectable fluorescent immunocomplex. With this method, the lower detection limit is 10.0 pg/mL for GFAP, 0.50 pg/mL for NfL, and 0.10 pg/mL for tau. The batch‐specific measurement range was for GFAP 50.8–28,200 pg/mL, for NfL 3.32–1556 pg/mL and for tau 0.50–271 pg/mL. All samples were analysed simultaneously, using the same batch of reagents, by board‐certified laboratory technicians who were blinded to the clinical data. The blood samples from the control group were re‐analysed de novo in the same batch as the samples from the AIS group to ensure comparable data unaffected by the analysis method. Intra‐assay coefficients of variation were < 10%.

### 2.4 Data Analyses and Statistics

As this was a pilot study, no formal sample size calculation was performed.

Descriptive statistics were used for patient characteristics. Data distribution was compared using the Shapiro–Wilk test and *Q*–*Q* plots. Data are presented as the median with interquartile range (IQR) or as the mean with standard deviation (SD). Comparisons between time points for each biomarker were performed using a related‐samples Friedman's two‐way analysis of variance by ranks test, and data for each time point are shown as median and IQR. For comparisons between the AIS and control groups, logarithmic transformation was applied and normality was reassessed prior to parametric testing. Group comparisons were made using a parametric test for independent samples (Student's *t*‐test) at each time point.

Missing data were not imputed. Analyses were performed using available data for each time point and biomarker.

A *p*‐value of 0.05 or less was considered statistically significant. All data are presented; no outliers are removed in any of the tables or figures. Extreme outliers were defined as > 3 IQR and mild outliers as 1.5–3 IQR, according to the SPSS boxplot criteria.

Sensitivity analyses were performed where all significant values were also tested without outliers and the results of the sensitivity analyses are reported in text.

Statistical analyses were performed using IBM SPSS Statistics for Windows, version 30.0.0. (IBM Corp. Armonk, NY, USA).

### 2.5 Artificial Intelligence Generated Content

Generative artificial intelligence Chat GPT was used for language editing.

## 3 Results

In the AIS group, 26 patients were eligible for sampling; however, in five of these, we could not obtain serial blood samples, as one patient died, two were transferred, and two withdrew consent. Therefore, data from 21 patients were analysed. In the control group, 23 patients were eligible for sampling; however, in four patients, there was insufficient material, resulting in data from 19 patients being analysed. One preoperative and two samples at 24 h were missing. In the AIS group, the mean age was 78.1 years, and 66.7% of participants were female. The mean age in the control group was 64.5 years, and 52.6% of participants were female. General anaesthesia was used for endovascular thrombectomy in 70% of patients in the AIS group, while 30% received sedation. In the control group, all patients received general anaesthesia. Contrast extravasation during the thrombectomy procedure was not observed in any of the patients. Successful recanalization was achieved in 20 of 21 patients. Furthermore, none of the patients in the AIS group had a postprocedural haemorrhagic transformation.

Baseline characteristics are presented in Table 1.

**TABLE 1:** Baseline characteristics.

		Acute ischaemic stroke *n* = 21	Controls *n* = 19
Age	Years mean (SD)	78.1 (11.3)	64.5 (11.4)
Sex	Female, *n* (%)	14 (66.7)	10 (52.6)
Comorbidities	Hypertension, *n* (%)	8 (38.1)	8 (42.1)
	Diabetes, *n* (%)	4 (19.0)	2 (10.5)
	Atrial fibrillation *n* (%)	7 (33.3)	0
	Congestive heart failure, *n* (%)	2 (9.5)	0
	Kidney disease	2 (9.5)	1 (5.3)
	Previous TIA, minor stroke/stroke	5 (23.8)	0
Time from *witnessed* stroke onset to thrombectomy, (WU strokes not included), minutes mean (SD)		104.8 (59.1)	
NIHSS^a^ mean (range)		12.3 (4–23)	
Wake‐up stroke *n* (%)		14 (66.7)	
Occluded vessel *n* (%)	M1 right	7 (33.3)	
	M1 left	4 (19.0)	
	M2 right	3 (14.3)	
	M2 left	3 (14.3)	
	A2 left	1 (4.8)	
	P1 left	1 (4.8)	
	Basilar	1 (4.8)	
	Carotid	1 (4.8)	

Abbreviations: A2, anterior cerebral artery Segment 2; Basilar, basilar artery; Carotid, carotid artery; M1‐2, middle cerebral artery Segment 1 or 2; *n*, numbers; NIHSS, National Institutes of Health Stroke Scale; P1, posterior cerebral artery Segment 1; SD, standard deviation; TIA, transient ischaemic attack; WU, wake‐up.

^a^ On arrival to the hospital, before intervention.

Table 2 presents the median and IQR for the biomarkers at the different time points for AIS and controls. All biomarkers showed significantly higher concentrations at 24 h compared with baseline, except for GFAP in the control group.

**TABLE 2:** Brain injury biomarkers in acute ischaemic stroke and controls, comparisons between time points for each biomarker and group.

Time		Before thrombectomy/surgery	2 h after thrombectomy/surgery	24 h after thrombectomy/surgery	*p*
GFAP pg/mL median (IQR)	Acute ischaemic stroke	301 (219)	292 (1460)	1443 (3264)	0.001
	Control	127 (111)	129 (113)	135 (96)	0.449
NfL pg/mL median (IQR)	Acute ischaemic stroke	25.5 (39.8)	26.2 (39.3)	38.3 (42.1)	0.001
	Control	12.9 (9.1)	15.7 (10.5)	19.8 (13.4)	0.001
Tau pg/mL median (IQR)	Acute ischaemic stroke	5.7 (3.7)	5.9 (3.1)	6.8 (4.3)	0.002
	Control	3.5 (2.7)	4.8 (3.6)	4.4 (1.3)	0.047

Abbreviations: GFAP, glial fibrillary acidic protein; IQR, interquartile range; mL, milliliter; NfL, neurofilament light chain; pg, picogram; tau, total tau.

Table 3 presents comparisons between the AIS group and controls for each biomarker at each time point using transformed natural logarithmic data. Compared with the control group, the AIS group showed significantly higher concentrations of GFAP and NfL at all time points, whereas tau concentrations were significantly higher only at 24 h.

**TABLE 3:** Brain injury biomarker concentrations (natural logarithm transformed)‐ comparisons between patients with acute ischaemic stroke and controls at each time point.

Group	Acute ischaemic stroke	Control	*p*‐value independent samples *t*‐test
ln GFAP mean (SD)			
Before thrombectomy/surgery	5.8 (1.0)	4.9 (0.5)	0.002
2 h after thrombectomy/surgery	6.2 (1.2)	4.8 (0.4)	0.001
24 h after thrombectomy/surgery	7.1 (1.3)	4.9 (0.5)	0.001
ln NfL mean (SD)			
Before thrombectomy/surgery	3.4 (0.9)	2.5 (0.5)	0.001
2 h after thrombectomy/surgery	3.4 (0.8)	2.5 (0.5)	0.001
24 h after thrombectomy/surgery	3.7 (0.7)	2.9 (0.5)	0.001
ln Tau mean (SD)			
Before thrombectomy/surgery	1.6 (0.5)	1.3 (0.5)	0.09
2 h after thrombectomy/surgery	1.7 (0.6)	1.6 (0.5)	0.6
24 h after thrombectomy/surgery	2.1 (0.8)	1.5 (0.4)	0.009

*Note:* Data are presented as mean (SD).

Abbreviations: GFAP, glial fibrillary acidic protein; ln, natural logarithm; NfL, neurofilament light chain; SD, standard deviation; tau; total tau.

These data are supported by boxplots for visualisation, (Figure 1a–c) for each of the biomarkers (median, IQR, range). In the boxplots *represents extreme outliers > 3 IQR, and 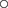 represent mild outliers 1.5–3 IQR, as per the definition by SPSS.

**FIGURE 1:**
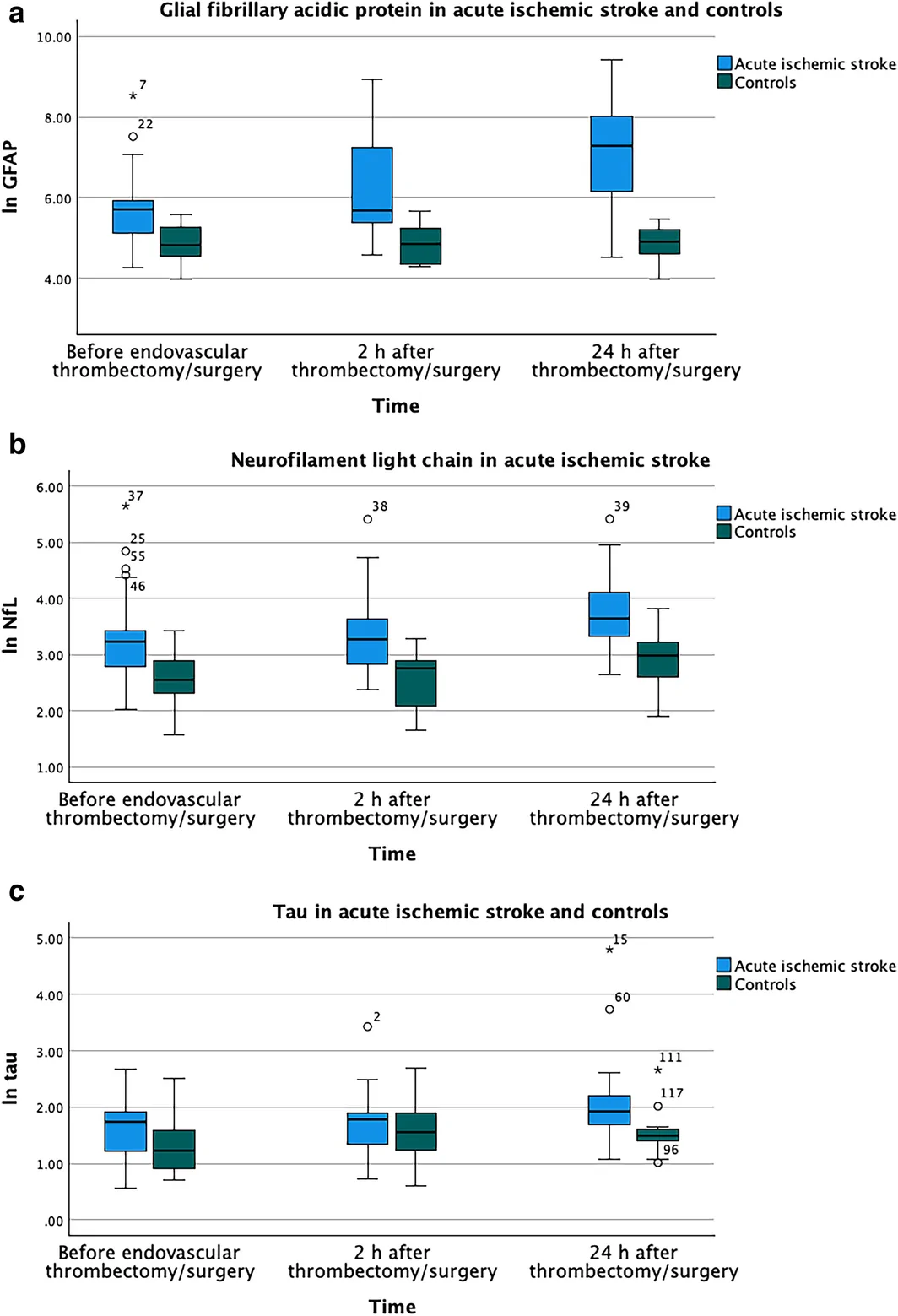
(a) Glial fibrillary acidic protein in acute ischaemic stroke and controls. Boxplots showing GFAP concentrations in patients with acute ischaemic stroke and surgical controls at the three sampling time points. (b) Neurofilament light chain in acute ischaemic stroke and controls. Boxplots showing NfL concentrations in patients with acute ischaemic stroke and surgical controls at the three sampling time points. (c) Tau in acute ischaemic stroke and controls. Boxplots showing tau concentrations in patients with acute ischaemic stroke and surgical controls at the three sampling time points.

### 3.1 Sensitivity Analyses

For GFAP, the difference between the AIS and control groups remained significant even after exclusion of the two outliers at time point 1 (before thrombectomy/surgery). For NfL, several outliers were present at time point 1; after their exclusion, the difference between groups was no longer statistically significant at time point 1 but remained significant at 2 and 24 h. For tau, the difference at 24 h was no longer statistically significant after exclusion of outliers.

## 4 Discussion

This study investigated circulating concentrations of GFAP, NfL and tau in patients with acute ischaemic stroke undergoing thrombectomy and compared them with surgical controls.

To approximate the clinical environment of the ICU and operating theatre, we restricted inclusion to patients undergoing endovascular thrombectomy as this procedure is commonly performed under sedation or general anaesthesia. This allowed us to study patients with ongoing cerebral ischaemia in a setting relevant for future clinical applications of biomarker‐based monitoring. The control group comprised patients without known neurological disease, undergoing surgery in general anaesthesia, with invasive monitoring and well‐preserved physiological parameters. This set‐up was chosen to approximate certain physiological conditions encountered in stable ICU patients.

The main results of this study are that blood biomarkers GFAP, NfL, and tau all displayed statistically significant increases in response to ongoing cerebral ischaemia. Additionally, the concentrations of all investigated biomarkers increased significantly from baseline to 24 h in the AIS group. When the AIS group was compared to the control group, a statistically significant difference was observed at all time points for biomarkers GFAP and NfL. However, for tau, the difference was not evident until 24 h after baseline samples, suggesting that GFAP and NfL respond more rapidly to an acute ischaemic event. For GFAP, a significant difference between the AIS group and controls was present at time point 1 and remained significant after exclusion of outliers in the sensitivity analysis, suggesting that GFAP may be a robust biomarker even in the early stages of the trajectory, a finding also supported by a recent study by Sjölin et al. [27] However, for NfL, the difference between the AIS group and controls was abolished at time point 1 after exclusion of outliers but remained at 2 and 24 h, suggesting a slightly slower trajectory than that of GFAP but a faster trajectory than that of tau.

The temporal increase in GFAP, NfL and tau observed after thrombectomy should also be interpreted in the context of reperfusion. While the biomarker release is likely driven primarily by ischaemic brain injury, successful recanalization may facilitate the passage of brain‐derived proteins into the systemic circulation through restoration of blood flow and reperfusion‐related blood–brain barrier disruption. Thus, the observed biomarker trajectories may reflect both ongoing release from injured tissue and enhanced washout following reperfusion. As all AIS patients in this study underwent endovascular thrombectomy, the present design does not allow differentiation between biomarker release caused by ischaemia per se and that attributable to reperfusion. Future studies comparing patients with successful and unsuccessful reperfusion, or serial sampling before and immediately after recanalization, may help clarify the relative contributions of these mechanisms.

In the search for a blood biomarker capable of detecting cerebral ischaemia in sedated or anaesthetised patients, or in patients with reduced consciousness, this pilot study suggests that GFAP may be a promising biomarker for further investigation.

GFAP increases significantly after cerebral ischaemia, but not in the controls. GFAP appears less affected by anaesthesia, sedation and surgery than both NfL and tau. Whilst NfL and tau display statistically significant increases in response to ongoing cerebral ischaemia, they also increase significantly in controls. This finding suggests that the elevation of NfL and tau in blood may be confounded by their expression in extracerebral tissues, complicating the interpretation in patients undergoing surgery, invasive interventions or those with traumatic injuries.

Brain‐derived tau is a brain‐specific biomarker now available for analysis [28]. Unfortunately, we did not have access to this more brain‐specific tau at the time of this study; thus, the analysis in the present study is on total‐tau.

This study has several limitations. The sample size in this pilot study is small, in both cohorts, which limits the generalizability of the findings. The variations in baseline values in the AIS group likely reflect differences in the time elapsed from stroke onset to the first sampling. Interpretation is complicated by the fact that 67% of the AIS group suffered from wake‐up stroke, and hence the exact time from stroke onset to first sampling could not be determined. Furthermore, the inclusion process of the AIS group may have introduced selection bias.

While these variations limit comparison of individual values, they provide a realistic insight into the potential difficulties of interpreting brain injury biomarker sampling in acute clinical settings. Furthermore, all the patients in the AIS cohort had large or medium vessel occlusions, causing significant ischaemic events; hence, it is unknown how smaller ischaemic events would affect these brain injury biomarkers. As our results indicate that at the last sampling time point (24 h after thrombectomy or surgery) the biomarker concentrations had not yet begun to decrease, a longer sampling timeline would have yielded more knowledge about the trajectory of brain injury biomarkers after AIS. Furthermore, we were unable to age‐ and sex‐match the two groups, which may affect the results, as brain injury biomarkers increase with age [29]. No adjustments were made for age, sex or multiple analyses, since this is a pilot study and the sample size is small. The findings should therefore be interpreted with caution. In this study, postprocedural neurological outcome measures such as NIHSS and mRS were not systematically collected, which limits our ability to relate biomarker trajectories to neurological recovery and functional outcome.

A limitation of the practical, clinical use of GFAP is that, according to previous publications, it is even more pronounced after haemorrhage than ischaemia [19]. In this study, no patients had haemorrhage; therefore, we cannot determine differences in GFAP levels between ischaemia and haemorrhage in anaesthetised or sedated patients. Five patients in the AIS group had previously had a transient ischaemic attack, minor stroke or stroke. Our data do not allow us to determine whether this affected the results; however, it should be considered when interpreting the findings.

In the AIS group, reflecting current clinical practice, 30% of patients were sedated and 70% had general anaesthesia. No statistically significant differences in biomarker concentrations were detected between patients receiving sedation and general anaesthesia; however, the small subgroup sizes preclude firm conclusions.

In the AIS group, anaesthesia was tailored to avoid hypotension to preserve cerebral perfusion to the ischaemic penumbra. In the control group, anaesthesia was tailored to the surgical procedure. Consequently, the anaesthetic procedures differed between the groups.

A biomarker for the detection of cerebral injury that is not affected by anaesthesia, sedation, surgery, or other interventions could be of considerable clinical value in many situations in anaesthesia and intensive care. Potential clinical applications include situations with compromised cerebral circulation such as hypotension from hypovolaemia or septic shock, but also after cardiac arrest, in prothrombotic conditions and in severe intoxication or metabolic conditions. Radiology is the gold standard in all these situations, but unstable patients and patients undergoing surgery may be unsuited. Further studies investigating the potential of blood‐based biomarkers in the ICU and operating theatre setting are needed to confirm or dismiss the findings of this pilot study.

## 5 Conclusion

In this pilot study, GFAP and NfL concentrations were consistently higher in patients with acute ischaemic stroke than in surgical controls, whereas tau differed only at 24 h. GFAP increased following stroke but not after non‐cerebral surgery, suggesting that it may be less affected by anaesthesia and surgical intervention than NfL and tau. These findings support further investigation of GFAP as a potential biomarker of acute cerebral injury in patients undergoing anaesthesia or intensive care, although larger studies are required to confirm its clinical utility.

## Author Contributions

R.V., L.B., H.H., and J.L. planned and designed the study. R.V., A.K., A.C., A.E.‐M., and P.M. collected data and obtained consent. K.J. and A.R. assessed patients neurologically, decided on, and performed the thrombectomies. H.Z. analysed samples and contributed to the methods section. C.B. and R.V. planned, collected, and handled samples. M.P. and L.B. made the statistical analyses. L.B., H.G., R.V., and J.L. drafted the manuscript. All authors revised the manuscript for important intellectual content, approved the final version, and agree to be accountable for all aspects of the work.

## Funding

The study was financed by Swedish governmental funding of clinical research (ALF) ALFGBG‐964972 and ALFGBG‐1005061, by Wilhelm and Martina Lundgren, Science foundation 2019‐3078 and 2021‐3677 and by The Gothenburg society of medicine GLS‐1022123 and GLS‐973013.

## Ethics Statement

The study was approved by the Swedish Ethical Review Authority (Dnr 2020‐00169) on 10‐03‐2020, with amendment 2020‐05122 on 2020‐10‐31 and 2023‐07506‐02 on 2024‐01‐23 and the protocol adheres to the latest version of the Declaration of Helsinki.

## Conflicts of Interest

The authors declare no conflicts of interest.

## Data Availability

The data that support the findings of this study are available from the corresponding author upon reasonable request.
